# Nine Lessons about Aquatic Invasive Species from the North Temperate Lakes Long-Term Ecological Research (NTL-LTER) Program

**DOI:** 10.1093/biosci/biae062

**Published:** 2024-08-26

**Authors:** M Jake Vander Zanden, Adrianna Gorsky, Gretchen J A Hansen, Pieter T J Johnson, Alexander W Latzka, Alison Mikulyuk, Robin R Rohwer, Michael J Spear, Jake R Walsh

**Affiliations:** Center for Limnology at the University of Wisconsin–Madison, Madison, Wisconsin, United States; Center for Limnology at the University of Wisconsin–Madison, Madison, Wisconsin, United States; Department of Fisheries, Wildlife, Conservation Biology at the University of Minnesota, Twin Cities, Minnesota, United States; Department of Ecology and Evolutionary Biology at the University of Colorado Boulder, Boulder, Colorado, United States; Wisconsin Department of Natural Resources, Madison, Wisconsin, United States; Aquatic Sciences Center at the University of Wisconsin–Madison, Madison, Wisconsin, United States; Department of Integrative Biology at the University of Texas at Austin, Austin, Texas, United States; Illinois River Biological Station, at the University of Illinois Urbana-Champaign, Havana, Illinois, United States; Department of Fisheries, Wildlife, Conservation Biology at the University of Minnesota, Twin Cities, Minnesota, United States

**Keywords:** long-term ecological research, distribution, abundance, invasive species, impact

## Abstract

Freshwater ecosystems can serve as model systems that reveal insights into biological invasions. In this article, we summarize nine lessons about aquatic invasive species from the North Temperate Lakes Long-Term Ecological Research program and affiliated projects. The lessons about aquatic invasive species are as follows: Invasive species are more widespread than has been documented; they are usually at low abundance; they can irrupt from low-density populations in response to environmental triggers; they can occasionally have enormous and far-reaching impacts; they can affect microbial communities; reservoirs act as invasive species hotspots; ecosystem vulnerability to invasion can be estimated; invasive species removal can produce long-term benefits; and the impacts of invasive species control may be greater than the impacts of the invasive species. This synthesis highlights how long-term research on a freshwater landscape can advance our understanding of invasions.

As a result of human activities, trade, and transport, species are increasingly establishing populations outside of their native range (Elton 1958, Lockwood et al. 2013, Seebens et al. 2017). Introduced species can subsequently spread, with large consequences for the recipient ecosystems (Strayer 2010, Ricciardi et al. 2013). Introduced species have resulted in ecological disruption, the loss of biodiversity, economic impacts, and reduced human well-being (Vitousek et al. 1996, Ricciardi and MacIsaac 2000, Pejchar and Mooney 2009, Strayer 2010). Hereafter, we consider a species that has established outside of its historical geographic range and that exerts (or, at least, has the potential to exert) undesired ecological or economic impacts to earn the label of *invasive species*.

Species invasions have proven to be especially problematic in freshwater ecosystems for several reasons (Ricciardi and MacIsaac 2000, Cox and Lima 2006, Havel et al. 2015, Moorhouse and Macdonald 2015, Gallardo et al. 2016). First, freshwater systems are already strongly degraded as a result of human activities, especially changing land-use and pollution owing to their downstream position within watersheds (Carpenter et al. 1998, Dudgeon et al. 2006, 2011). Second, many freshwater systems are patches of habitat embedded in a sea of land. Such low levels of habitat connectivity leads to geographic isolation and a high degree of endemism, such that freshwater ecosystems are biodiversity hotspots (Dudgeon et al. 2006, Reid et al. 2019), especially given that freshwater habitats are a tiny fraction of the Earth's surface. However, there are an estimated 117 million lakes worldwide (Verpoorter et al. 2014), placing the grand challenge of addressing the issue of aquatic invasive species in perspective (Lodge et al. 2006). Finally, it is clear that invasive species and their impacts cannot be considered in isolation from other interacting drivers of environmental change, such as climate change, land-use change, hydrologic alterations, and nutrient pollution (Rahel and Olden 2008, Strayer 2010, Carpenter et al. 2011).

Given both the scope and the impacts of freshwater species invasions, there is a pressing need to develop an integrated understanding of their spread, establishment, biotic interactions, and ecosystem consequences (Strayer 2010, Ricciardi et al. 2013) in order to inform and guide their management (Lodge et al. 2006). Such an integrated understanding will not emerge from any single approach or type of study. Rather, insights emerge from a combination of approaches: experimental manipulations, long-term studies, cross-system comparisons, theory, and modeling. In this article, we summarize nine lessons about freshwater invasive species that have emerged over the past two decades from the North Temperate Lakes Long-Term Ecological Research (NTL-LTER) program. One component of the NTL-LTER program is long-term monitoring of a series of lakes going back to 1981 (Magnuson et al. 2006, Carpenter et al. 2007). We note that the NTL-LTER program is much more than long-term sampling of 11 lakes. It is a question-driven program that integrates multiple approaches for understanding environmental change. Long-term sampling of the core NTL-LTER lakes has undoubtedly provided insights into freshwater species invasions and into the role of invasive species as agents of ecosystem change at decadal time scales. In addition, insights have come from several spatially extensive cross-lake studies that have elucidated broadscale patterns by virtue of their spatial coverage. It also includes whole-ecosystem experiments that can help reveal mechanistic relationships (Carpenter et al. 1995). We note that NTL-LTER investigators have also led many closely affiliated spinoff projects that are intellectually linked to the NTL-LTER program. In the present article, we summarize insights from this body of work in the form of nine key lessons about the ecology and management of invasive species (table 1). Our lessons apply across different stages of the biological invasion process (Vander Zanden and Olden 2008)—how aquatic invasive species arrive, survive, and affect ecosystems, as well as their management. Several of these insights challenge conventional assumptions about invasive species and have direct implications for managing freshwater biodiversity and ecosystems.

**Table 1.** Nine lessons about invasive species from research affiliated with the North-Temperate Lake Long-Term Ecological Research (NTL-LTER) program in a lake-rich region, Wisconsin, in the United States.

Lesson	References
Invasive species are more widespread than has been documented	Vander Zanden et al. 2017
Invasive species are usually at low abundance	Hansen et al. 2013c
Invasive species can irrupt from low-density populations in response to environmental triggers	Spear et al. 2021b
Invasive species can occasionally have enormous and far-reaching impacts	Walsh et al. 2016a
Invasive species can affect microbial communities	Rohwer et al. 2023a
Impoundments act as invasive species hotspots and stepping stones	Johnson et al. 2008
Invasive species vulnerability assessments can inform management	Vander Zanden and Olden 2008
Invasive species removal can produce ecosystem shifts and long-term benefits	Lathrop et al. 2013, Perales et al. 2021
The impacts of invasive species control may be greater than the impacts of invasive species	Mikulyuk et al. 2020b

## Study system

**Figure 1.**
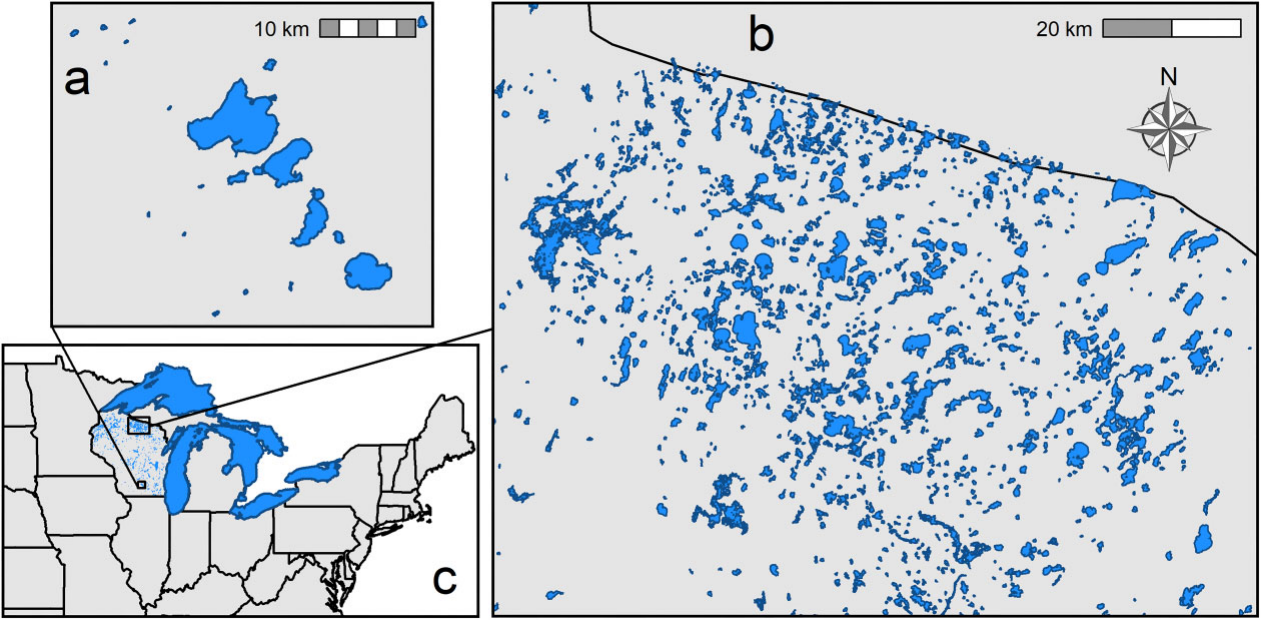
Map showing (a) the Yahara Lake District and (b) the Northern Highlands Lake District, both part of the North Temperate Lakes Long-Term Ecological Research (NTL-LTER) site, and (c) the broader regional context of the upper Great Lakes region of North America, including the Laurentian Great Lakes.

In this article, we focus on research from the past two decades on the ecology of aquatic invasive species conducted as part of the NTL-LTER research program and affiliated projects. This site of the US Long Term Ecological Research Network was launched in 1981. Originally, the core study lakes consisted of seven lakes in the Northern Highlands Lake District (NHLD) in northern Wisconsin, in the United States (figure 1). In 1994, four additional lakes from the Yahara Lake District (YLD) in Madison, Wisconsin, were added. Together, these two lake districts span a geographic and land-use gradient that is broadly representative of the lake-rich upper Great Lakes region that includes the states of Wisconsin, Michigan, and Minnesota (figure 1). Both lake districts were glaciated during the most recent Wisconsin glaciation (Martin and Hanson 1965). The moraines and glacial debris deposited by the receding glaciers created an irregular and undulating landscape that resulted in an astounding number of lakes. The Wisconsin Department of Natural Resources maintains a registry of inland waterbodies, which lists nearly 15,000 lakes, and the adjacent glaciated regions of Minnesota and Michigan include many thousands more.

The NHLD is known for having one of the highest densities of lakes in the world (Martin and Hanson 1965, Kratz et al. 1997, Magnuson et al. 2006). The lakes in the region vary widely with regard to geological setting, water chemistry, morphology, trophic state, and biotic composition (Magnuson et al. 2006, Carpenter et al. 2007). The NHLD was subject to intensive clearcut logging in the late nineteenth century. Today, the region is largely covered by second growth forest, and lakeshore residential development and disturbance of riparian zones are extensive and a major driver of environmental change. Lakes in the NHLD today are characterized by forested watersheds, low dissolved ion and nutrient concentrations (low conductivity), and high water quality.

The YLD is located at the southern glacial boundary of the Wisconsin glaciation (figure 1), and the lakes in this region were formed as the result of morainal damming. The land in the YLD watershed was cleared for agriculture in the midnineteenth century, and the urbanized area of Madison has expanded continuously ever since. The lakes in the YLD have agricultural and urbanized watersheds, high dissolved ion concentrations, and high nutrient levels. Agricultural and urban nutrient runoff lead to nuisance algal blooms and poor water quality.

Both lake districts have been affected by the introduction of nonnative species from a broad range of taxa (Vander Zanden and Maxted 2008, Escobar et al. 2018). Nonnative species introductions date back to the late 1800s, when common carp (*Cyprinus carpio*) were purposefully introduced into the YLD lakes. More recent examples include Eurasian watermilfoil (*Myriophyllum spicatum*), which established and spread in the region during the 1970s, zebra mussel (*Dresseina polymorpha*) in the 1990s and 2000s, and spiny water flea (*Bythotrephes cederstroemi*) in the 2000s (Vander Zanden and Maxted 2008), all presumably accidental introductions.

An important feature of our two lake districts and the study region generally is its proximity to the Laurentian Great Lakes (figure 1). The Great Lakes have acted as a beachhead for the arrival of invasive species that have subsequently undergone secondary spread to inland waters (Rothlisberger and Lodge 2013). The construction of the St. Lawrence Seaway in the 1950s opened the Great Lakes to oceanic vessels from around the world, and ballast water transport is responsible for many high-impact invasive species into the lakes (Mills et al. 1994, Ricciardi and MacIsaac 2000, Holeck et al. 2004). The Great Lakes are among the most invaded freshwater ecosystems in the world, with at least 184 nonnative species (Mills et al. 1993, 1994, Ricciardi and MacIsaac 2000). Human activity (bait buckets, recreational boating) has facilitated the gradual spread of a subset of these species from the Great Lakes to inland waters (Bossenbroek et al. 2001, Vander Zanden and Olden 2008, Rothlisberger et al. 2010, Kelly et al. 2013). These secondary invasions from the Great Lakes have affected the ecology and economies of inland lakes and provide a model landscape for understanding invasive species arrival, spread, and impact, yielding lessons and insights that can be broadly applied.

## Lesson 1: Invasive species are more widespread than has been previously documented

Distribution and geographic range are among the most fundamental features of any species’s ecology. Landscape-level or broadscale studies of species, including invasives, often present a map of the species’s geographic range. The geographic range represents the geographic area occupied by a species (Brown 1995, Gaston and Blackburn 2000, Gaston 2003) but does not convey critical finer-scale information about species occurrence and prevalence within the range—in other words, the amount and spatial distribution of potential habitat and habitat occupancy within that range (Thiele et al. 2010, Latzka et al. 2016, Vander Zanden et al. 2017).

The distinction between geographic range and prevalence and occurrence within the range is particularly important when considering species that inhabit discrete habitats such as lakes (Latzka et al. 2016, Vander Zanden et al. 2017). Lakes are well-defined habitat patches embedded within a predominantly terrestrial landscape and are therefore convenient, mostly isolated units in which one can assess and track invasions. Perhaps not surprisingly, there are numerous invasive species databases that document invasive species occurrences (Simpson et al. 2009). Aquatic invasive species occurrence records are also collected and maintained by many natural resource management agencies. For the state of Wisconsin, the Wisconsin Department of Natural Resources maintains an inventory of waterbodies known to contain aquatic invasive species (https://apps.dnr.wi.gov/lakes/invasives/AISByWaterbody.aspx). These data are updated regularly and are an important information source and management tool.

Knowledge of invasive species’ locations and their overall prevalence is critically important for informing lake management decisions (Bobeldyk et al. 2015, Vander Zanden et al. 2017). Imagine two hypothetical invasive species with similar invaded ranges, but one is much more widely distributed within that range. If all else is equal, the management and prevention strategies are likely to be very different; managing the less prevalent species may aim for containment within current lakes, whereas the strategy for the more prevalent species would entail shielding remaining uninvaded lakes (Drury and Rothlisberger 2008). At a more basic level, efforts to reduce or stop the spread of invasive species require information about where those species occur.

The existing inventory of invaded waterbodies for the state of Wisconsin provides an opportunity to evaluate how well a regional occurrence database reflects actual invasive species occurrence. Wisconsin's infested waterbody data set is populated with invasive species presence records from diverse information sources, including citizen science, biological monitoring, incidental reports from biologists and scientists, and records from invasive species research projects. More recently, there have been formal surveys aimed at detecting invasive species, although these are a small portion of the total records (Latzka 2015). Given the approximately 15,000 lakes in Wisconsin, an ongoing census of all lakes would be impossible. Because the list represents confirmed occurrences, one might expect that false presence measures (occurrence records where, in fact, the species is absent) would be infrequent. On the other hand, this is a presence-only database; therefore, we would expect that some invasive species occurrences have not been detected or reported and are, therefore, not included (Latzka 2015, Vander Zanden et al. 2017). Given the heterogeneous data sources, we might also expect gaps and biases associated with the available data. For example, small and inaccessible lakes may not be well represented, and lesser-known invasive species may be less thoroughly reported (Dickinson et al. 2010).

Invasive species prevalence from the infested waterbody list was compared with results from multiyear invasive species field surveys using a stratified random design and statistical weighting (Schade and Bonar 2005, Latzka 2015, Vander Zanden et al. 2017). The researchers surveyed 458 lakes for the presence of a suite of aquatic invasive species. The lake selection involved stratifying according to conductivity, presence or absence of a public boat launch, and lake size. From the infested waterbody list, the percentage of lakes for which one or more of six target invasive species (rusty crayfish, spiny water flea, dreissenid mussel, Chinese mystery snail, banded mystery snail, Eurasian watermilfoil) presence was summarized. Occurrence records from the existing infested waterbody list indicate that about 8% of Wisconsin lakes contain one or more of these six target invasive species (1189 lakes were listed as containing one or more of these species, out of 14,364 total lakes; Latzka 2015). The randomized field surveys of lakes indicated invasive species prevalence was much higher. Of 458 lakes surveyed, 338 contained one or more invasive species (Latzka 2015). Applying statistical weighting to the strata, it was estimated that 39% of Wisconsin lakes harbor at least one of these aquatic invasive species. This estimate is nearly five times higher than inferences based strictly on existing occurrence records (Latzka 2015). This difference was greater for poorly known species (e.g., Chinese mystery snails) than for high-impact species, such as zebra mussels. Considering that the state of Wisconsin has a strong infrastructure for collecting and documenting invasive species occurrences, our results suggest that at broad spatial scales, knowledge of aquatic invasive species distribution is poor (Bobeldyk et al. 2015). The degree of underestimation is likely to vary widely among regions and species. Although, of course, targeted surveys can characterize distributions for localized regions, collecting such information at broad spatial scales is likely to be prohibitive. Our understanding of the basic macroecology of invasive species—foundational to invasive species management—will continue to be limited by a paucity of occurrence data (Vander Zanden et al. 2017).

## Lesson 2: Invasive species are usually at low abundance

Much of our understanding of biological invasions is dichotomous in nature, with a strong emphasis on invasive species occurrence or presence or absence (lesson 1). For example, there has been a strong focus in invasion biology on predicting which species are likely to be invasive and cause ecological or economic harm (Kolar and Lodge 2002) and predicting sites that are vulnerable or likely to be invaded. Invasive species monitoring and databases generally focus on occurrence and not abundance. Although it is self-evident that invasive species abundance will vary among sites and through time, we know surprisingly little about spatial patterns of invasive species abundance. Spatial patterns of invasive species abundance are the subject of lesson 2.

**Figure 2.**
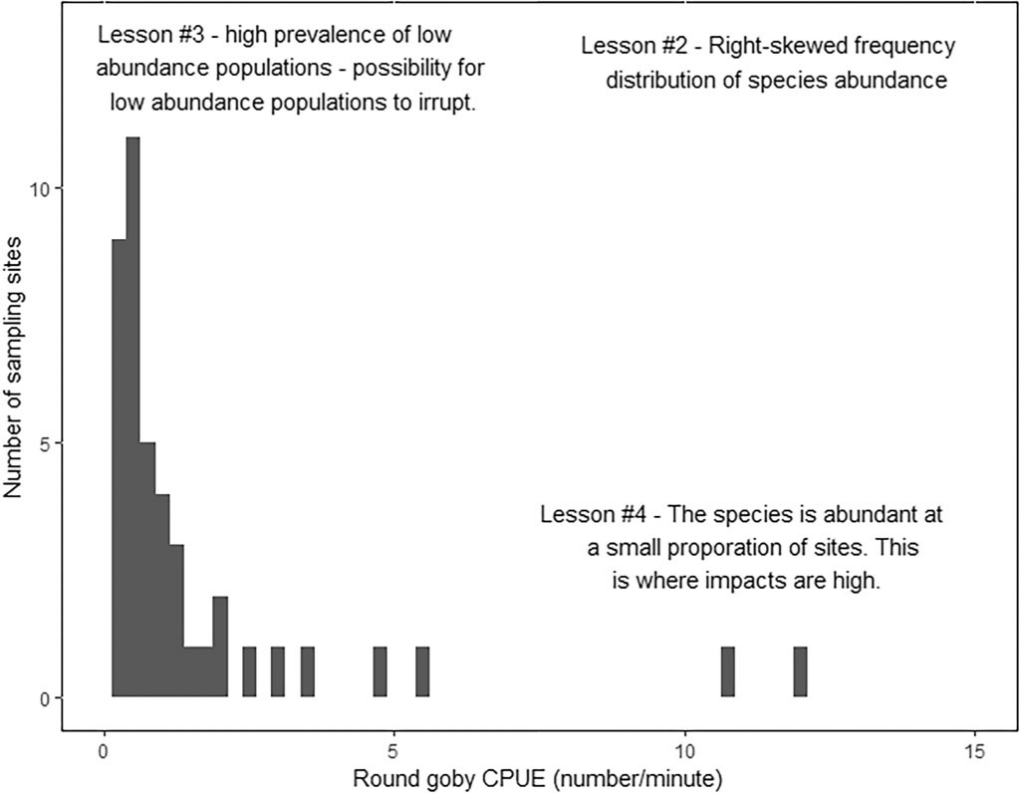
Frequency histogram showing spatial variation in invasive species abundance. To illustrate, we use the example of invasive round goby (Neogobius melanostomus; catch per unit effort, CPUE; the number of individuals captured per minute backpack shocking) from 60 Wisconsin streams. This plot helps to illustrate lessons 2, 3, and 4. Lesson 2 is that the frequency distribution of abundance for round goby is highly right skewed; that is, at most sites, the population occurs at low abundance. Lesson 3 is that, given that round goby are typically found at low abundance, we could imagine that there are additional sites in which round goby are present but were not detected in the field survey (i.e., were below the detection threshold). These detected and undetected low abundance populations create potential for population irruptions of this invasive species in response to environmental change or other possible triggers. Lesson 4 is that the invasive is at high abundance at a small number of sites (i.e., the right tail of the distribution). Given that ecological and economic impact increases with abundance, we expect high levels of impact to occur at the small number of high-abundance sites.

A core tenet of ecology is that most species at a given site occur at low abundance, whereas only a handful are abundant. These so-called species-abundance distributions are described as right skewed or log normal. Fewer studies have examined the abundance distribution of a single species across sites, although the same pattern holds; a given species is typically rare and achieves high abundance at only a few locations (Preston 1948, Brown 1995, Brown et al. 1995). Little work has addressed whether this basic pattern also holds true for invasive species (Labra et al. 2005). Hansen and colleagues (2013a) explicitly examined the abundance distributions of aquatic invasive species and found that they are often present at low abundance and infrequently reach high abundance (figure 2). The overall abundance distribution patterns were similar to those of native species, which were examined in the same study. This finding was somewhat surprising, given the common views that invasive species typically take over and that invasive species are sometimes even defined as species that typically reach high abundance. This finding challenges the conventional wisdom that invasive species show fundamentally different ecological patterns than native species and that invasives tend to be abundant where they occur (Hansen et al. 2013a, Vander Zanden et al. 2017).

Given the obvious interest in understanding invasive species effects, a key question is how local invasive species abundance is related to ecological or economic impact. As a general rule, invasive species impacts tend to increase with abundance. But the relationship between abundance and impact can take multiple forms (Yokomizo et al. 2009, Jackson et al. 2015), and it is possible for invasive species to produce strong impacts even at low abundance. Indeed, in some cases, the negative effects of invasive species may be highest at low or moderate densities (Kornis et al. 2014, Bradley et al. 2019). Unfortunately, the form of this abundance–impact relationship is rarely known (Sofaer et al. 2018). Taken together, it is the spatial distribution of the invasive species, the spatial pattern of abundance, and the form of the abundance–impact relationship that determine the overall impact of a species at the landscape scale. Variation in these details can result in vastly different cumulative impacts of invasive species when considered at broad scales (Thiele et al. 2010, Latzka et al. 2016). Understanding the landscape-level impacts of aquatic invasive species involves knowing where an invasive occurs, where it is abundant, and where it is impactful. These factors tend not to be well understood for most species (Vander Zanden et al. 2017). Given these knowledge gaps, understanding invasive species abundance, how it varies across the landscape, and the relationship between abundance and impact is critical for prioritizing and managing aquatic invasive species.

Given that invasive species frequently occur at low levels of abundance, there is a need for improved methods to detect low-abundance populations. Environmental DNA (eDNA) has boomed in popularity and shows promise for improving the sensitivity and efficiency of species detection in aquatic systems (Jerde et al. 2011, Goldberg et al. 2016). The utility of eDNA for detecting low-abundance populations of nonnative species may be useful although highly context dependent. eDNA monitoring of two low-density, nonnative species in NTL-LTER and nearby lakes yielded mixed detection efficiencies, likely driven by variable eDNA shedding rates of the target species and physical characteristics of the study system (Dougherty et al. 2016, Walsh et al. 2019). Consideration of the life history and density of target species should guide the timing and sampling effort to improve detection using eDNA and traditional detection methods (De Souza et al. 2016). Detecting extremely low-density populations in large search areas—even with advanced technologies—will likely to continue to be challenging (Walsh et al. 2018b).

## Lesson 3: Environmental triggers may cause low density populations of invasive species to irrupt

Populations of nonnative species may persist for long periods at low abundance, with self-sustaining reproduction but inconspicuous impacts on the recipient ecosystems. These low-abundance populations can undergo population irruptions, often suddenly, after persisting at low levels for decades (Spear et al. 2021b). The discovery of a nonnative zooplankter, the spiny water flea (*Bythotrephes cederstroemi*), in the YLD's Lake Mendota helps to illustrate this lesson. Spiny water flea appeared in Lake Mendota at remarkably high densities in fall of 2009. At first, it was assumed that the species had very recently been introduced. But subsequent analysis of sediment cores and museum specimens revealed that this population had likely persisted below the detection limit for at least a decade prior to its irruption and subsequent detection. Despite routine zooplankton monitoring in this lake, a sleeper population had gone undetected for years until environmental conditions (in this case, an anomalously cool summer) allowed it to flourish and establish a massive egg bank that continued to sustain a high abundance of spiny water flea going forward (Walsh et al. 2016b).

Sleeper populations of invasive species may irrupt when a change in the environment triggers an abrupt shift in invasive species abundance and impact. Although the early stages of any population expansion account for some inherent lags between a species introduction and an outbreak to high abundance (Crooks and Soulé 1999, Crooks 2005), it may be common that an environmental trigger causes an abrupt shift in abundance or impact. Triggers may include shifts in food-web dynamics, such as when a prey is added or a predator removed from the system, or the completion of an interrupted mutualism when a coevolved species is later introduced (Spear et al. 2021b). Triggers may also be environmental drivers that cross a threshold, either gradually (e.g., global warming) or stochastically (e.g., a heavy rain event). These triggers remove previous population constraints and allow abrupt population growth, causing a low-abundance and possibly undetected sleeper population to become a full blown, invasive nuisance. Examples of sleeper populations have been documented from around the world (Spear et al. 2021a). In the northern Wadden Sea (Germany), nonnative cordgrass (*Spartia anglica*) persisted at low densities since the 1920s despite cold water temperatures. In the past few decades, however, the earlier onset of spring has caused water temperatures to more often exceed the critical temperature thresholds for successful germination and photosynthesis, increasing cordgrass production and spread (Loebl et al. 2006). As another example, on Macquarie Island (Australia), prey-limited cats (*Felis catus*) received a resource boost from introduced rabbits, allowing the cats’ numbers to grow and their diet to expand to include native birds (Courchamp et al. 1999). A growing list of examples support the idea that low-abundance (and undetected) populations may be widespread across the landscape, representing a stockpile of potential sleeper populations awaiting environmental triggers. In an era defined by global environmental change, this potential buildup may represent a major challenge for invasive species management. Many practical questions remain: How often do low-abundance populations irrupt? Are invasive species irruptions sometimes only temporary? Do certain species or ecosystem traits associate with certain triggers? Can we manage sleeper populations by increasing resilience and other forms of ecosystem management that reduce the risk of triggers? How can we improve the detection of low-abundance populations before they irrupt? Can we distinguish new invasions as a recent introduction from irruption from a sleeper population? This work highlights the potential for unaccounted-for invasion debt in the form of widespread but low-abundance populations of invasive species on landscapes. Whether this invasion debt eventually manifests as costly ecological impacts will be determined not so much by efforts to stop invasive species spread but, rather, by the stressors and conditions in those ecosystems that trigger sleeper population outbreaks (Spear et al. 2021b).

## Lesson 4: Invasive species impacts can occasionally be enormous and far reaching

Lessons 2 and 3 were focused on the left side of the species-abundance histogram shown in figure 2. For lesson 4, we shift our focus to the right side of the abundance histogram—that is, the long tail of the abundance distribution. Note that, in figure 2, the invasive round goby occurs at high abundance in a very small fraction of the surveyed sites. This spatial pattern of species abundance (lesson 2) is fundamentally important to our understanding of invasive species impacts at the landscape scale (Thiele et al. 2010, Latzka et al. 2016, Vander Zanden et al. 2017). An invasive species’s impact generally increases as a function of abundance, although the abundance–impact relationship can take on several possible forms, including linear, sigmoidal, and threshold responses (Yokomizo et al. 2009, Jackson et al. 2015, Latzka et al. 2016). Nevertheless, a key implication is that the small fraction of ecosystems in which invasives become very abundant are the ecosystems in which we expect that the level of impacts will be high and, moreover, that highly affected systems are expected to be relatively uncommon (Latzka et al. 2016, Vander Zanden et al. 2017).

To illustrate the enormous impacts that are possible when invasive species reach high densities, we consider again the example of the invasive spiny water flea. This zooplankton species was reported in inland lakes of the region starting in the early 2000s. The levels of abundance were generally low, and no notable impacts were reported (although invaded lakes were not well studied at the time). In fall of 2009, the spiny water flea was detected in an NTL-LTER core sampling lake, Lake Mendota, Wisconsin. The population reached enormous densities, at times exceeding 1000 individuals per cubic meter. Annual average densities exceeded 100 individuals per cubic meter, which were the highest densities reported for North America (Walsh et al. 2016a). The spiny water flea invasion of Lake Mendota represented the long tail (right side) of the abundance distribution (figure 2).

The ecological impacts of spiny water flea in Lake Mendota were striking. Spiny water fleas are predatory on other zooplankton, including *Daphnia pulicaria* (hereafter, *Daphnia*), which is a keystone grazer that supports clear water by grazing on algae. Following the spiny water flea invasion, overall predation pressure on zooplankton more than doubled (Walsh et al. 2017). *Daphnia* biomass declined by over 90%, the algal biomass increased, and the water clarity decreased by 1 meter (Walsh et al. 2016a, 2017).

The water clarity in the Madison lakes supports recreational and aesthetic ecosystem services that are valued highly by citizens in the region (e.g., the average willingness to pay for improved water quality was US$353 in 2001 per household; Stumborg et al. 2001, Walsh et al. 2016a). Many past efforts to improve water quality have centered on reducing the input of nutrients that fertilize algae growth (Lathrop et al. 1998, Lathrop 2007, Lathrop and Carpenter 2014). In the late 1980s, a food-web biomanipulation was attempted to improve the water quality in Lake Mendota. Piscivorous fishes walleye (*Sander vitreus*) and northern pike (*Esox lucius*) were stocked at high levels. This effort to shift the food web to piscivore dominance happened to correspond with a major die-off of zooplanktivorous lake herring (*Coregonus artedii;* Vanni et al. 1990, Kitchell 1992, Rudstam et al. 1993). The consequence was a sharp increase in *Daphnia* and an increase in water clarity of approximately 1 meter (Lathrop et al. 2002). The low zooplanktivore, high *Daphnia* ecosystem state persisted in Lake Mendota for several decades thereafter but was undone by the spiny water flea, which reverted Lake Mendota to an ecosystem state similar to that before the biomanipulation (Walsh et al. 2017).

Given that there are limited management options for an invasive zooplankton once it has established, a key question was how this loss of water quality could be alleviated through other means. The obvious approach for improving water clarity in the YLD is a reduction of external phosphorus loading. Notably, NTL-LTER research provided the basis for understanding the relationship between phosphorus loading and water clarity before and after the spiny water flea invasion. From this, it was estimated that the clarity of the water before introduction of the spiny water flea could be attained with a 70% reduction in external phosphorus loading. Programs aimed at nutrient reduction have been implemented in the Lake Mendota watershed for decades, although phosphorus loading has remained approximately constant. Using published estimates of the cost of various nutrient reduction strategies, it was estimated that a 70% reduction in phosphorus loading would cost between US$86.5 million and US$163 million (Walsh et al. 2016a), which corresponds with the willingness to pay estimate from Stumborg and colleagues (2001).

The spiny water flea example illustrates how species invasions can sometimes produce wholesale shifts in ecosystem state (e.g., cascading impacts that extend to the base of the food web and water quality), highlighting the potential for invasive species to produce exceptionally high levels of impact in specific situations. It also provides an example of how invasive species impacts can be expressed in terms of ecosystem services; in this case, two different methods converged to produce an estimate of approximately US$100 million of economic impact for the Lake Mendota spiny water flea invasion. This impact is for a single invasive species in a single lake. But as was noted earlier, the high levels of abundance and adverse impacts of spiny water flea documented in Lake Mendota appear to be the exception. Spiny water fleas are at low to moderate levels of abundance elsewhere and appear not to have produced notable impacts on water clarity in other invaded lakes. In a Minnesota lake, simultaneous invasions of spiny water fleas and zebra mussels produced a net zero effect on water quality (Rantala et al. 2022). Trout Lake, in the NHLD, is a possible exception to the above; spiny water fleas invaded in 2014 and produced a decrease in water clarity (Martin et al. 2022), although notably, in the last few years, the spiny water flea population in Trout Lake has collapsed, and the impact on water quality appears to have reversed, which is consistent with the concept of a boom–bust dynamic (Strayer et al. 2017).

The spiny water flea invasion of Lake Mendota illustrates that invasive species can sometimes have profound impacts on ecosystems. But this situation appears not to be the norm, and in many cases, abundance is low and impacts are minimal (see lesson 2). We speculate that the Lake Mendota ecosystem was especially primed for spiny water flea impacts. In Lake Mendota, planktivorous fishes were at low abundance, and *Daphnia*, a favored prey, was at high abundance and played an important role in maintaining water clarity (a role that was revealed by the spiny water flea invasion). The combination of these factors may have promoted both spiny water flea establishment and large ecological impact in this ecosystem (Walsh et al. 2016b).

## Lesson 5: Invasive species can affect microbes

Aquatic microbes are complex, interconnected communities that change over space and time. Microbial communities respond to lake physiochemical characteristics (Somers et al. 2019, Paver et al. 2020), land-use change and eutrophication (Rozmarynowycz et al. 2019, Kraemer et al. 2020), and seasonal change (Ávila et al. 2017, Zhu et al. 2019). In Lake Mendota, microbial communities exhibit strong seasonal patterns (Shade et al. 2007, Rohwer et al. 2023a). Given the central role of microbes in regulating biogeochemical processes (Cotner and Biddanda 2002), a key knowledge gap has been how species invasions affect microbes (cyanobacteria and heterotrophic bacteria) and associated microbe-mediated processes.

Species invasions can affect the seasonal dynamics and abundance of harmful microbes such as cyanobacteria. Looking to Lake Mendota as an example, the phytoplankton follow the traditional Plankton Ecology Group model for a eutrophic lake: a spring diatom bloom followed by a summer dominated by cyanobacteria, with diatoms reemerging in fall (Carey et al. 2016). These overall patterns did not shift following spiny water flea invasion in 2009, but the spring diatom bloom increased in magnitude and persisted for longer (Walsh et al. 2018a). Some minor components of the spring bloom, such as green algae, increased alongside diatoms, whereas others, such as cryptophytes, remained constant (Rohwer et al. 2023b). The most notable change in the spring phytoplankton communities was an earlier seasonal onset of cyanobacteria. Historically, the clearwater phase denoted a transition between a diatom-dominated and cyanobacteria-dominated community. However, after the spiny water flea invaded, cyanobacteria began appearing at the start of clearwater phase, and after the zebra mussels invaded, they began appearing before clearwater phase (Rohwer et al. 2023a).

The spiny water flea invasion did not change the overall summer cyanobacteria biomass, but the cyanobacteria’s diversity increased, perhaps in response to a narrowing grazing pressure from reduced zooplankton diversity (Rohwer et al. 2023a). Although the Lake Mendota zebra mussel invasion (2015) did not lead to shifts in summer cyanobacteria abundance, composition, or diversity, shifts in summer cyanotoxins were observed. Absolute concentrations of the hepatotoxin microcystin increased in early summer, and the duration that toxins were observed in the lake was extended by 53 days (Rohwer et al. 2023a). This illustrates the complex implications of microbial change; cyanotoxin production changed even though cyanobacteria did not.

The Spiny water flea and zebra mussel invasions also affected the heterotrophic microbial community. With the exception of the Bacteroidota phylum, most bacterial responses were distinct between closely related taxa and were specific to certain seasons (Rohwer et al. 2023a). Overall, the two invasions differed in their seasonal impacts. The spiny water flea had the greatest effects on bacteria in spring and clearwater, whereas the zebra mussels had a more even impact across seasons, with the exception of late summer, when very few bacteria changed in abundance (Rohwer et al. 2023a). This was notable because late summer is also the consistently toxic period of the lake, and the cyanotoxin phenology did change in response to the zebra mussels.

The microbial loop, by recycling nutrients and carbon, serves to connect the food web with biogeochemical cycles. Therefore, microbes can link changes in the food web with changes in physiochemical processes. For example, the spiny water flea triggered a trophic cascade that increased and lengthened the spring diatom bloom (Walsh et al. 2018a). This resulted in higher organic matter deposition early in the growing season, which was degraded by bacteria. This consumed hypolimnetic oxygen, such that anoxia onset began 2 weeks sooner following stratification (Rohwer et al. 2023b). Although the roles of individual microbes are often poorly understood, the impact of changes in the microbial community can be far reaching, affecting lake-management-level concerns such as toxins and fish habitat.

## Lesson 6: Impoundments act as invasive species hotspots and stepping stones

Not all ecosystems are equally vulnerable to species invasion. In the present article, we highlight NTL-LTER work showing that impoundments (lentic waterbodies resulting from dam building) are more likely than natural lakes to support one or more aquatic invasive species. In a survey of 1080 waterbodies in Wisconsin, five aquatic invasive species—the zebra mussel, the spiny water flea, the rusty crayfish, the rainbow smelt, and the Eurasian watermilfoil—were 2.4 to 7.8 times more likely to occur in impoundments than in natural lakes (Johnson et al. 2008). Among 189 waterbodies surveyed for the three most common invaders, impoundments were also significantly more likely to support multiple aquatic invasive species concurrently (Johnson et al. 2008). Impoundments can similarly serve as a source population for aquatic invasive species to spread into nearby natural lakes and aquatic habitats (Liew et al. 2016, Anas and Mandrak 2021, Comte et al. 2021, Hedden et al. 2021, Pfauserová et al. 2021).

The status of impoundments as hotspots for invasive species likely owes to both an increased likelihood of species arrival and introduction and high levels of environmental suitability (Havel et al. 2005). In terms of arrival, impoundments are more likely to be accessible to humans and to have more extensive hydrological connections, both of which increase exposure to invader propagules. In the Wisconsin study, for instance, impoundments were 68% more likely to be accessible and had 4.3 times more boat landings (a measure of access and boating intensity) and a watershed 44.6 times larger than natural lakes (Johnson et al. 2008). The physical and biological characteristics of impoundments can also increase environmental suitability. In contrast to most natural lakes, impoundments tend to be younger in age and have lower species richness, leading to more available niches and weaker biotic resistance to species introductions. Impoundments also tend to be more disturbed (i.e., artificial fluctuations in water levels) and more productive (Havel et al. 2005, Johnson et al. 2008), both of which are conducive to invasive species establishment (Davis et al. 2000, Kolar and Lodge 2001, Strayer 2010).

Looking forward, an important challenge is to understand the degree to which impoundments function as invasion hubs or stepping stones that facilitate secondary spread into natural lakes. Using a spatial analysis that incorporated typical distances traveled by Wisconsin boaters, Johnson and colleagues (2008) found that the construction of impoundments increased the number of natural lakes that are vulnerable to introductions from nearby invaded systems (see also Havel et al. 2015). Impoundments may also act as evolutionary stepping stones; because of their high salinity compared with natural lakes, impoundments near coastal environments offer intermediate habitats to facilitate colonization by marine taxa that subsequently adapt to freshwater (Lee 2002, Havel et al. 2005). Similarly, the tropical cladoceran *Daphnia lumholtzi* has spread rapidly in impoundments across the midwestern USA and, more recently, in South America (Nunes et al. 2021). It often shows higher densities in or near impoundments and can persist at colder temperatures, possibly highlighting adaptive change. These observations underscore the importance of examining how human habitat modification (in this case hydrologic alterations) may facilitate invasive species spread, and the broader issue of the interactive effects of these two important drivers of environmental change (Didham et al. 2005, 2007).

## Lesson 7: Ecosystem vulnerability to invasion can be estimated

Given the ongoing spread of invasive species and their potential to produce impacts, resource management agencies have invested heavily in programs and strategies aimed at stopping or slowing invasive species spread. Moreover, there is recognition that understanding ecosystem vulnerability to species invasion can help guide and inform decisions about allocating limited management effort and resources for “stop the spread” campaigns (Vander Zanden and Olden 2008). This matter is highly relevant in lake-rich regions that are being invaded by multiple invasive species, such as the NTL-LTER study region (Vander Zanden and Olden 2008). As such, NTL-LTER has addressed conceptual and practical aspects of ecosystem vulnerability to invasive species.

Two broad factors determine the vulnerability of an ecosystem to invasive species: those necessary for a species to arrive and those required to survive (Leung and Mandrak 2007, Vander Zanden and Olden 2008). Arrival gets at the transport and introduction of invasive species propagules (Riccardi 2006, Hulme 2009, Havel et al. 2015). Given that there are many possible pathways and vectors that can lead to species introduction, this tends to be highly species specific. For inland lakes, lakeshore homeowners and the lake-to-lake movement of recreational boaters in particular are important vectors for invasive species spread (Rothlisberger et al. 2010, Kao et al. 2021, Ashander et al. 2022). Variables such as the presence or number of boat launches, lake size, and the degree of residential lakeshore development are indicative of the risk of introduction and are predictors of invasions (Johnson et al. 2008, Olden et al. 2011). Furthermore, in the multilake invasive species field surveys described in lesson 1, we noted that lakes lacking road access and residential development were also completely free of invasive species (Latzka 2015). This further supports the notion that human activities are a key driver of invasive species occurrence (Strayer 2010, Havel et al. 2015). In contrast, survival relates to a species’s fundamental niche and whether an ecosystem provides suitable habitat for a given species (Jeschke and Strayer 2008, Vander Zanden and Olden 2008, Kulhanek et al. 2011). This environmental matching is critical; repeated introductions of a species into an environment that is outside of the fundamental niche of that species will not result in species establishment.

NTL-LTER researchers have used a variety of modeling approaches to identify vulnerable systems and forecast the spread of aquatic invasive species in the region. Mercado-Silva and colleagues (2006) developed a statistical model predicting the presence or absence of the rainbow smelt in lakes in the species’s native range in Maine, which was assumed to be saturated. The study subsequently used the model to identify suitable lakes in other regions, identifying 553 environmentally suitable lakes for the rainbow smelt in Wisconsin. Similar analyses have been developed for other invasive species of management concern in the study region. Papes and colleagues (2016) used maximum entropy modeling to identify Wisconsin lakes that are environmentally suitable for Chinese mystery snail. Dissolved calcium concentrations were the basis for identifying environmentally suitable lakes for the zebra mussel (Papes et al. 2011). Olden and colleagues (2011) estimated the likelihood of introduction and establishment for the invasive rusty crayfish. Mikulyuk and colleagues (2020a) developed a model predicting both occurrence and abundance for the invasive Eurasian watermilfoil. All of the above studies developed statistical associations between the occurrence of known invasive species populations and environmental factors and subsequently used this as a basis for identifying the degree of ecosystem vulnerability and risk of future invasion in uninvaded systems (Vander Zanden and Olden 2008). In practice, measuring invasive species abundance across a lake-rich landscape is resource intensive and often intractable. Models for predicting invasive species abundance from environmental conditions can be useful for identifying locations likely to have high abundance and are, therefore, most likely to be affected by invasive species (Kulhanek et al. 2011, Mikulyuk et al. 2020a).

## Lesson 8: Invasive species removal can produce ecosystem shifts and long-term benefits

Given the impacts and societal concern over invasive species, a great deal of effort has been invested in developing approaches for invasive species control (Escobar et al. 2018). Complete eradication of an invasive population is sometimes the goal but can be exceptionally difficult and costly (Myers et al. 2000). Nevertheless, eradication can be achieved in isolated habitats such as islands or ponds, and the long-term benefits of localized eradication in certain circumstances can be quite clear (Myers et al. 2000, Jones et al. 2016). What is much less well known is under what circumstances invasive species control can produce meaningful and lasting environmental or economic benefits and to what extent the benefits outweigh the costs (Epanchin-Niell and Wilen 2012, Green and Grosholz 2021). A general concern is that the benefits of invasive species control may be transient, in that it alleviates undesired impacts in the short term, but the invasive species population simply rebounds when control efforts cease (Zipkin et al. 2009). We still know little about under what conditions invasive species control is likely to produce long-term benefits.

Ecologists have learned a great deal about the potential for ecosystems to undergo transitions between alternative ecosystem states and the general nature of abrupt ecosystem shifts (Scheffer et al. 2001, Scheffer 2009, Carpenter 2003). A central question is whether invasive species removal can shift an ecosystem into an alternative (preferred) ecosystem state and, importantly, whether the new state is persistent over time (Scheffer et al. 2001, Hansen et al. 2013b). If so, it would not only indicate the potential for abrupt ecosystem shifts but also that even short-term invasive species removal could produce persistent benefits (Ratajczak et al. 2018).

In the present article, we summarize insights from two ecosystem experiments indicating that short-term invasive species removal can produce rapid ecosystem shifts and lasting benefits (Perales et al. 2021). The first was in Lake Wingra, Wisconsin, a 140-hectare, shallow lake (with a maximum depth of 3.8 meters) within the YLD. As an urban lake, it receives significant amounts of nutrient-rich stormwater runoff and has experienced frequent algal blooms and impaired recreational value. The lake has abundant Eurasian watermilfoil and a substantial population of common carp (*Cyprinus carpio*). The common carp is known to resuspend bottom sediments and nutrients during feeding, thereby increasing algal blooms and turbidity. It is widely accepted that the presence of the common carp can maintain an ecosystem in a turbid state through a series of reinforcing feedback loops (Bajer and Sorensen 2015). It was therefore hypothesized that sharply reducing common carp through a removal program would trigger a positive feedback and an ecosystem shift to a clearwater, macrophyte-dominated state (Lathrop et al. 2013).

**Figure 3.**
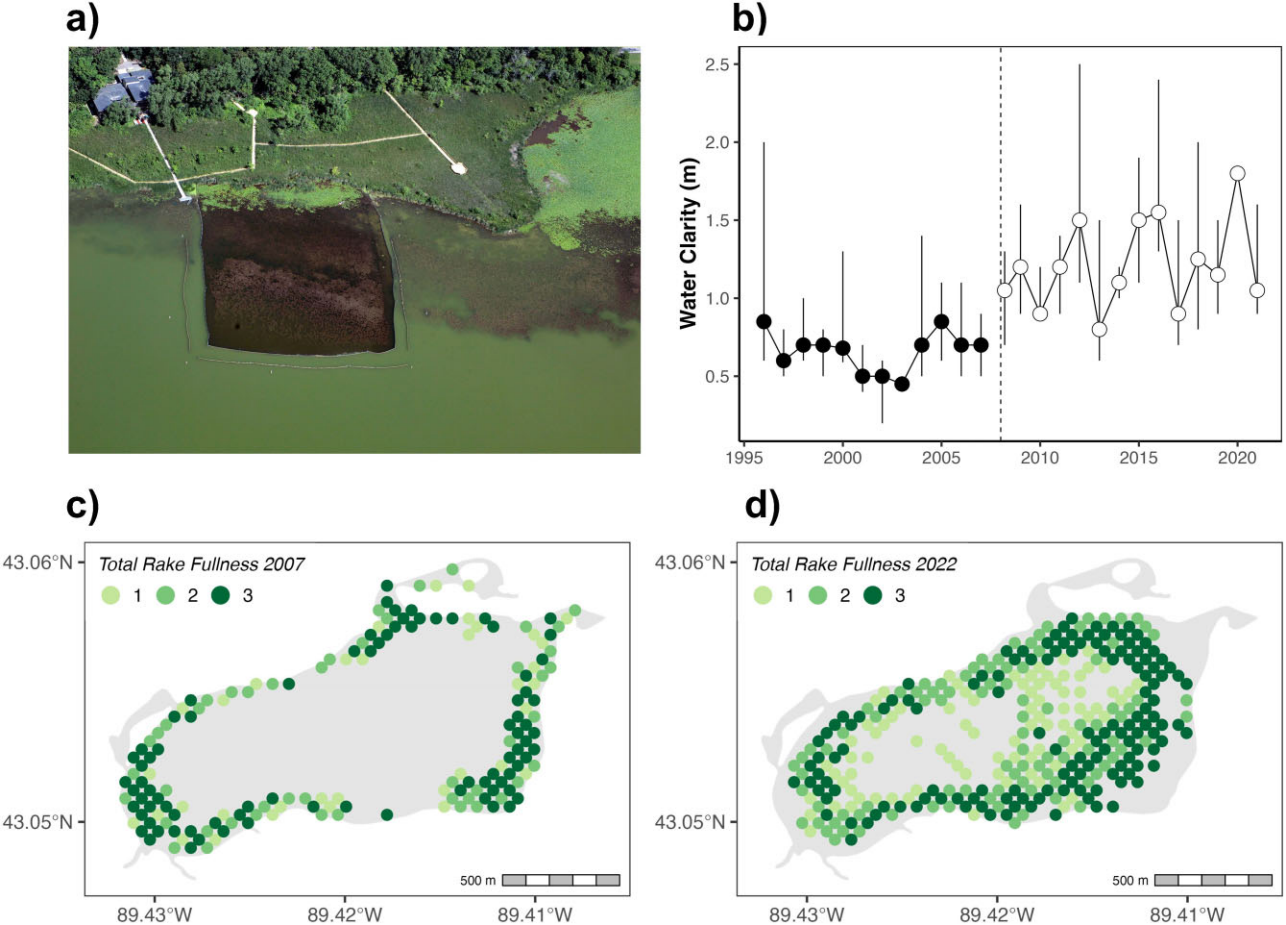
Changes in Lake Wingra in response to invasive carp removal. (a) A carp exclosure experiment in Lake Wingra showing much clearer water within the 1-hectare exclosure, in stark contrast with the turbid conditions in the lake (Lathrop et al. 2013). Photograph: Mike DeVries, 7 July 2007. (b) Median summer (June–August) water clarity (the vertical lines show the minimum and maximum values) measured by Secchi disk in Lake Wingra over the period 1995–2020. The dashed vertical line indicates the March 2008 carp removal. (c) Macrophyte abundance in Lake Wingra based on rake fullness in 2007, before carp removal. (d) Macrophyte abundance in Lake Wingra based on rake fullness in 2022, following carp removal. The shift to the clearwater state corresponded with a sharp increase in macrophyte cover and biomass.

As a first step, a 1-hectare carp exclosure was installed in Lake Wingra in 2005 (Lathrop et al. 2013). The following year, water clarity increased within the exclosure relative to the surrounding lake (figure 3a), and the native aquatic plants responded positively to this increase in clarity. Building from these promising results, a commercial fisher was hired to remove carp through the ice in March of 2008. The fisher removed 23,600 kilograms of carp. In the following years, the water in Lake Wingra was notably clearer (figure 3b; Magnuson et al. 2023), and beach closings due to algal blooms became less frequent. Aquatic plants (both native and nonnative Eurasian watermilfoil) increased in terms of cover, expanding into deeper waters (figure 3c and 3d). This study revealed that a one-time removal of the invasive common carp shifted this lake from a turbid to a clearwater or macrophyte-dominated state (Lathrop et al. 2013). Moreover, this new ecosystem state has persisted for approximately 15 years. A key question is why the common carp population did not simply rebound following this short-term removal. One hypothesis is that predation by native fishes such as bluegill (*Lepomis macrochirus*) on juvenile common carp and carp eggs has prevented the population of carp from rebounding (Bajer et al. 2012).

The second ecosystem experiment involved the removal of the invasive rusty crayfish (*Faxonius rusticus*) from Sparkling Lake, in the NHLD. This clearwater 64-hectare lake was invaded by the rusty crayfish in the early 1980s. This aggressive invasive crayfish reached high levels of abundance and produced declines in native sunfish and native virile crayfish (*Faxonius virilis*; Hansen et al. 2013c). Intensive rusty crayfish removal using crayfish traps began in 2001 (Hein et al. 2006, 2007). In conjunction, fisheries managers placed limits on the recreational harvest of gamefishes known to consume adult crayfish. Over 8 years of rusty crayfish removal, catch rates of rusty crayfish declined by more than 95%, whereas native crayfish and sunfish gradually rebounded. The rusty crayfish removal led to a shift in state that was ecosystem-wide (Hansen et al. 2013b, 2013c). A key question was whether the system exhibited alternative stable states (i.e., multiple stable equilibria possible under identical conditions, *sensu* May 1977) or, alternatively, that the shift was the result of a simple threshold response. Time-series models were not able to clearly resolve these two possibilities (Hansen et al. 2013c). Nevertheless, the desirable low rusty crayfish state has persisted for nearly 15 years after the cessation of the rusty crayfish removal (Perales et al. 2021). From a practical perspective, a key finding is that intensive crayfish removal produced a desirable ecosystem shift and that this shift was remarkably persistent over time.

Although efforts to control nuisance invasive species are on the rise, it is rare that invasive species control is coupled with long-term data collection and used to test basic ideas about alternative stable states and abrupt ecosystem shifts (Strayer et al. 2006). As a result, our understanding of the long-term ecosystem effects of invasive species and the benefits and consequences of invasive species control is limited. The long-term data of NTL-LTER provided a critical context for ecosystem experiments designed to improve our understanding of how ecosystems respond to management. In both examples in the present article, invasive species removal produced dramatic ecosystem shifts that persisted. These examples show that invasive species removal can be a valuable strategy for ecological restoration. Of course, invasive species removal projects will not always produce persistent ecosystem-wide shifts. A key question is under what conditions such shifts occur.

## Lesson 9: Impacts of invasive species control may be greater than the impacts of invasive species

Invasive species removal can greatly reduce invasive species abundance and induce desirable ecosystem changes that are persistent (Perales et al. 2021). However, it is essential to consider invasive species removal in a broader ecosystem context. Invasive species removal can produce indirect, cascading food-web effects (Zavaleta et al. 2001), and the use of pesticides in control can produce nontarget effects. Given that invasive species control could have unanticipated consequences and could pose a risk to ecosystems and nontarget organisms, it is important to consider when and whether the benefits of invasive species control outweigh the risks and potential harm.

The use of herbicides to control aquatic invasive plants in lakes is a common practice in North America (Nault et al. 2018). Lake-wide chemical treatments can result in long exposure times and have been linked to unintended lethal and sublethal effects on lake biota. For example, large-scale 2,4-D treatments were conducted in Lake Ellwood, Wisconsin, to control the hybridized Eurasian watermilfoil (*Myriophyllum spicatum*) annually over 10 years (Schleppenbach et al. 2022). Although chemical treatment reduced plant abundance, it was also linked to a decline in zooplankton and recruitment failures of important gamefish, such as the largemouth bass (*Micropterus salmoides*) and the bluegill (*Lepomis macrochirus*). After treatments ceased, the recruitment of game fish and zooplankton abundance rebounded. In other studies, large-scale herbicide treatment led to a decrease in water clarity and declines in native aquatic plant species (Wagner et al. 2007, Nault et al. 2014, Kujawa et al. 2017, Nault et al. 2018).

A large, cross-lake synthetic study of lakewide herbicide treatments to control invasive Eurasian watermilfoil in Wisconsin lakes showed a link between lakewide herbicide treatment and declines in aquatic plant abundance and shifts in community composition (Mikulyuk et al. 2020b). Notably, the effects of herbicide treatment were larger and more negative than those associated with the Eurasian watermilfoil itself—the invasive species that was the target of the treatment (Mikulyuk et al. 2020b). This result highlights the trade-offs involved and the need to carefully consider both the impacts and the benefits of invasive species control, especially when using methods that have negative effects on nontarget organisms.

Although there are certainly cases of invasive species being successfully eradicated from an aquatic system, the examples are rare. Managers typically attempt eradication in circumstances where conditions are favorable—for example, species that are conspicuous or in small, isolated ecosystems. There is also impetus to attempt eradication early in the invasion process to halt establishment and spread of new colonists (Vander Zanden et al. 2010). Most often, however, invasive species control projects are intended to minimize undesired local impacts. In the case of controlling invasive aquatic plants, the benefits of invasive species control are often short term. In the above example where herbicide treatment harmed the fishery (Schleppenbach et al. 2022), it was initially hoped that, following the initial treatment, the managers could shift to small-scale herbicide and mechanical control. Unfortunately, the invasive Eurasian watermilfoil population rebounded, exceeding the capacity of small-scale intervention.

A key conclusion is that there is no one-size-fits-all approach to invasive species control. Although all large-scale ecosystem interventions should consider the potential costs and benefits, not all management actions pose the same degree of risk. The above highlights the need for risk analysis frameworks that weigh multiple factors—the magnitude of invasive species impact, the price tag for control, and the potential for unexpected or nontarget impacts (Vander Zanden et al. 2010). Another key lesson is that the promise of silver bullet solutions should be approached with skepticism. Lessons emerging from invasive species control successes and failures call on us to test, verify, and adapt, using data-driven strategies that acknowledge uncertainty.

## Conclusions

The overarching goal of this article was to synthesize key insights and lessons pertaining to aquatic invasive species that have emerged from the NTL-LTER program. Although the signature long-term data collection of this LTER site was a component for the research we have summarized, these insights reflect a combination of long-term studies, ecosystem experiments, comparative studies, and modeling. This study is an example of how broad and synthetic insights emerge through integration of insights derived from multiple approaches.

### Are there general lessons to be derived from the study of invasive species in lakes?

We aimed to summarize lessons that have potential to be general in nature and have broad applicability. Moreover, much of this work is rooted in basic ecological concepts such as species distribution and spatial patterns of abundance. We recognize that the insights and lessons summarized in the present article derive exclusively from research conducted in lakes in one geographic region. A key question is to what extent these lessons are applicable to other contexts and ecosystem types. Other lake districts will undoubtedly differ with regard to which invasive species are spreading, the specific pathways and vectors of spread, and ecosystem attributes that affect ecosystem vulnerability. So although the details may unfold differently in other lake districts, our hope is that this article touches on concepts and ideas that are still of interest. More broadly, streams and terrestrial habitats tend to be more open systems than lakes. Terrestrial habitats also tend to have fewer distinct boundaries than lakes do. For example, in dealing with lakes, a binary classification of invasive species (i.e., presence or absence) has proven to be a useful, albeit imperfect, construct. In more open ecosystem types, the concept is less readily applied, although in many cases, it may still be applied—for example, if we consider habitat patches in terrestrial ecosystems (Thiele et al. 2010).

### Scaling up, heterogeneity, and landscape ecology

Several of our lessons, specifically lessons 1–4, were tightly interconnected; we considered spatial patterns of invasive species occurrence and found that invasive species tend to be more widespread than has typically been documented. We also considered the spatial patterns of invasive species abundance and found that invasive species often occur at relatively low abundance. Of course, this is not always the case, and under certain circumstances, where invasive species reach high levels of abundance, they can have dramatic impacts and produce ecosystem-wide shifts. A key finding is that invasive species abundance and impact are spatially heterogeneous. We should not expect invaded ecosystems to be universally affected.

### Invasives in a changing world

It is critical to recognize that ecosystems are not static and that ecosystems are currently undergoing change as a result of climate change, as well as other anthropogenic drivers (O'Reilly et al. 2015). Temperature is a critically important dimension of a species’s niche (Magnuson et al. 1979, Magnuson and Destasio 1996). In response to climate warming, the range of certain invasive species is expected to shift northward, enabling further expansion into new areas or expanding the number of suitable systems in a region (Rahel and Olden 2008, Thomas 2010). In lakes, climate change may result in a shift in the depths at which suitable thermal habitat occurs, pushing some species into deeper waters that may or may not be habitable, depending on oxygen levels (Kraemer et al. 2021). Warming could also reduce the suitability of ecosystems to certain invasive species, leading to a reduction in the species’s invaded range (Walsh et al. 2020). For others, warming could trigger existing sleeper populations to irrupt. How invasive species respond to climate change will be highly species specific; we simply highlight that ecosystem states are not static; rather, they are undergoing change stemming from multiple drivers of global environmental change (O'Reilly et al. 2015).

Species introductions are undoubtedly a major driver of environmental change for inland water ecosystems. Given the geographic isolation and distinct boundaries of many aquatic systems, the introduction or irruption of a nonnative species can have huge effects and can produce abrupt ecological shifts. For this reason, lakes provide useful model systems for studying biotic change, and examining these changes in lakes can help us better understand the drivers and consequences of ecological change in a general sense. This work not only has environmental management implications but highlights our still-evolving understanding of the distribution, abundance, impact, and management of invasive species in ecosystems. Our work also highlights the critical challenge in ecology of scaling up from detailed work done on a small number of local sites to a landscape or a region (Lodge et al. 1998). Ecosystems are remarkably heterogeneous, and how ecosystems respond to a driver is highly context dependent. Efforts to scale up local ecosystem-specific work to broader spatial scales will continue to be an important goal in understanding the spread and impact of invasive species.

## Author Biography

M. Jake Vander Zanden (mjvanderzand@wisc.edu) and Adrianna Gorsky are affiliated with the Center for Limnology at the University of Wisconsin–Madison, in Madison, Wisconsin, in the United States. Gretchen J. A. Hansen and Jake R. Walsh are affiliated with the Department of Fisheries, Wildlife, and Conservation Biology at the University of Minnesota, Twin Cities, Minnesota, in the United States. Pieter T. J. Johnson is affiliated with the Department of Ecology and Evolutionary Biology at the University of Colorado Boulder, in Boulder, Colorado, in the United States. Alexander W. Latzka is affiliated with the Wisconsin Department of Natural Resources, in Madison, Wisconsin, in the United States. Alison Mikulyuk is affiliated with the Aquatic Sciences Center at the University of Wisconsin–Madison, in Madison, Wisconsin, in the United States. Robin R. Rohwer is affiliated with the Department of Integrative Biology at the University of Texas at Austin, in Austin, Texas, in the United States. Michael J. Spear is affiliated with the Illinois River Biological Station, at the University of Illinois Urbana-Champaign, in Havana, Illinois, in the United States.
